# Phorbol Esters Isolated from *Jatropha* Meal Induced Apoptosis-Mediated Inhibition in Proliferation of Chang and Vero Cell Lines

**DOI:** 10.3390/ijms131113816

**Published:** 2012-10-24

**Authors:** Ehsan Oskoueian, Norhani Abdullah, Syahida Ahmad

**Affiliations:** 1 Department of Microbiology, Faculty of Biotechnology and Biomolecular Sciences, Universiti Putra Malaysia, 43400, Serdang, Selangor, Malaysia; E-Mail:ehs424@yahoo.com; 2 Agriculture Biotechnology Research Institute of Iran (ABRII)-East and North-East Branch, P.O.B. 91735/844, Mashhad, Iran; 3 Department of Biochemistry, Faculty of Biotechnology and Biomolecular Sciences, Universiti Putra Malaysia, 43400, Serdang, Selangor, Malaysia; E-Mail: syahida@biotech.upm.edu.my; 4 Institute of Tropical Agriculture, Universiti Putra Malaysia, 43400, Serdang, Selangor, Malaysia

**Keywords:** phorbol esters, *Jatropha curcas*, *Jatropha* meal, proliferation, toxicity, apoptosis, flow-cytometry, DNA fragmentation, western blot, infrared imaging system

## Abstract

The direct feeding of *Jatropha* meal containing phorbol esters (PEs) indicated mild to severe toxicity symptoms in various organs of different animals. However, limited information is available on cellular and molecular mechanism of toxicity caused by PEs present in *Jatropha* meal. Thus, the present study was conducted to determine the cytotoxic and mode of action of PEs isolated from *Jatropha* meal using human hepatocyte (Chang) and African green monkey kidney (Vero) cell lines. The results showed that isolated PEs inhibited cell proliferation in a dose-dependent manner in both cell lines with the CC_50_ of 125.9 and 110.3 μg/mL, respectively. These values were compatible to that of phorbol 12-myristate 13-acetate (PMA) values as positive control *i.e.*, 124.5 and 106.3 μg/mL respectively. Microscopic examination, flow cytometry and DNA fragmentation results confirmed cell death due to apoptosis upon treatment with PEs and PMA at CC_50_ concentration for 24 h in both cell lines. The Western blot analysis revealed the overexpression of PKC-Δ and activation of caspase-3 proteins which could be involved in the mechanism of action of PEs and PMA. Consequently, the PEs isolated form *Jatropha* meal caused toxicity and induced apoptosis-mediated proliferation inhibition toward Chang and Vero cell lines involving over-expression of PKC-Δ and caspase-3 as their mode of actions.

## Introduction

*Jatropha curcas* Linn. belongs to the *Euphorbiaceae* family. Currently, this plant has gained importance as the *Jatropha* seeds have been recognized to be a potential source of oil for biofuel production [1]. Upon oil extraction, a byproduct called *Jatropha* meal which is high in protein is produced. *Jatropha* meal has the potential as a feed ingredient for the animal industry [2]. However, the presence of phorbol esters (PEs) in some genotypes limits its applications as a feed constituent. Toxicity of *Jatropha* meal has been reported in different animals such as goats, sheep, mice, rats and fish [3,4]. Direct feeding of *Jatropha* meal containing PEs indicated mild to severe symptoms in various organs of different animals, like hemorrhage in the gastrointestinal tract, kidneys, spleen and heart; enteritis, congestion, lung edema and excessive fluid in serous cavities [5].

Phorbol ester is a member of the tigliane family of diterpenes. They are polycyclic compounds in which two hydroxyl groups on neighboring carbon atoms are esterified to fatty acid [6]. The phorbol-12-myristate 13-acetate (PMA) is one of the PEs found in croton plant (*Euphorbiaceae*) and often employed in biomedical research to activate the signal transduction of protein kinase C (PKC) enzyme. Binding of PEs is the first step in the activation of PKC. This binding is saturable and occurs through specific interactions within the C1 domain in the regulatory region of the PKC molecule [7].

The PEs biological activities are strictly structure-dependent based on the functional groups present. Thus, different PEs may activate different PKC isozymes in the cells which could trigger different pathways leading to various responses including tumor formation, inflammation, differentiation and apoptosis in the animal tissues [8,9]. Hatton *et al*. [8] reported that PEs activate PKC-β I and II which regulate cells differentiation, PKC-ɛ which appears to regulate the expression of anti-apoptotic genes and PKC-Δ which is involved in both differentiation and apoptosis with the participation of caspase-3 protein.

Although the toxicity of the PEs present in *Jatropha* meal has been addressed previously, the mode of action of PEs from *Jatropha* meal still requires further investigation. Therefore, this research was conducted to determine the toxic effects and the mode of action of PEs isolated from *Jatropha* meal using human hepatocyte (Chang) and African green monkey kidney (Vero) cell lines.

## 2. Results and Discussion

### 2.1. Isolation of Phorbol Esters

**Figure 1:**
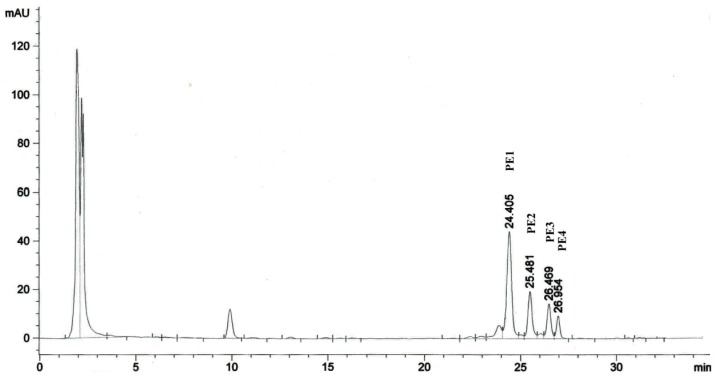
High-performance liquid chromatogram of phorbol esters (PEs) present in *Jatropha* meal.

High performance liquid chromatography (HPLC) analysis of methanolic extract obtained from *Jatropha* meal showed four peaks at the mentioned retention times (Section 3.2) and they were named as PE1, PE2, PE3 and PE4 (Figure 1). These components which represented the PEs of *Jatropha* meal, have been reported by Makkar *et al*. [10], Li *et al*. [11] and recently by Devappa [4]. However, Hass *et al*. [12] characterized six PEs in *J. curcas* seeds, namely Jatropha factor C_1_–C_6_ which possessed the same diterpene moiety identified as 12-deoxy-16-hydroxyphorbol. The isolated PEs observed appeared to be different in their dicarboxylic acid moieties (bicyclo[3.1.0]hexane or cyclobutane unit) and to be C-8 epimers. In a recent study, Roach *et al*. [13] also characterized the PE1, PE2, PE3 and PE4 and reported them as Jatropha factor C_1_, C_2_, C_3_ mixture and C_4_ + C_5_ mixture, respectively. In the present study, the four fractions of PEs isolated were pooled since the focus was to evaluate the toxic effects of PEs present in Jatropha meal. The concentrations of the isolated PEs used in this study were expressed as equivalents to the standard phorbol-12-myristate 13-acetate (PMA). The total yield of PEs isolated from Jatropha meal was 3 mg PMA equivalent/g dry matter of *Jatropha* meal.

### 2.2. Proliferation Assay

**Figure 2:**
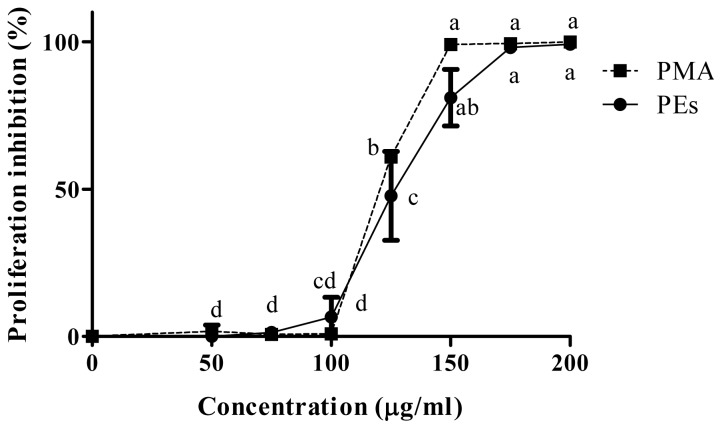
Percentage cells proliferation inhibition of isolated PEs and phorbol 12-myristate 13-acetate (PMA) on Chang cell line. The mean values (*n* = 3) at different concentrations with different superscripts differ significantly (*p* < 0.05).

**Figure 3:**
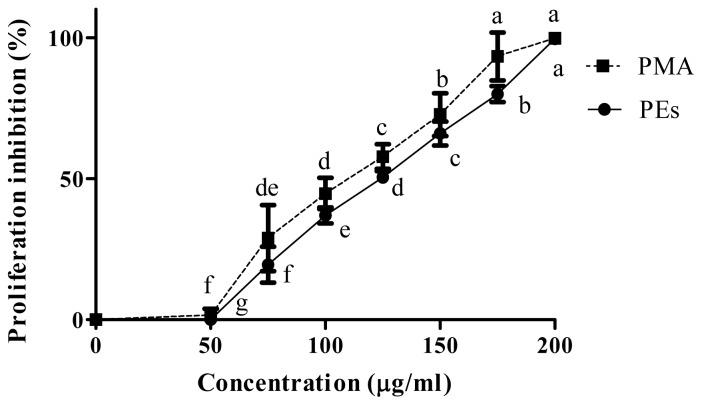
Percentage cells proliferation inhibition of isolated PEs and PMA on Vero cell line. The mean values (*n* = 3) at different concentrations with different superscripts differ significantly (*p* < 0.05).

The anti proliferation activity of isolated PEs and PMA as positive control in Chang and Vero cell lines are shown in Figures 2 and 3 respectively. Isolated PEs and PMA inhibited the cells proliferation significantly (*p* < 0.05) in a dose-dependent manner. In contrast, the Chang cells were less prone to the presence of PEs and PMA as compared to the Vero cells and the proliferation was only affected at 100 μg/mL and above. Mentlein *et al*. [14] and Goel *et al*. [9] previously reported the presence of phorbol ester hydrolyzing enzymes in the rat and mice liver such as acylcarnitine hydrolase and diacylglycerol lipase that degrade the PEs to their safe derivatives. Therefore, this resistance in Chang cells may be attributed to the presence of these enzymes.

The CC_50_ values presented in Table 1 show similar concentrations of PEs and PMA to inhibit the proliferation of 50% of the Chang and Vero cell lines. However, the CC_50_ values for Chang cells were significantly (*p* < 0.01) higher than that for Vero cells indicating the higher susceptibility of the cells to PEs and PMA. This result was in agreement with Li *et al*. [11] who reported the higher susceptibility of the lung and kidney when compared to other organs when mice were fed with phorbol ester-containing oil.

### 2.3. Microscopic Examination

**Figure 4:**
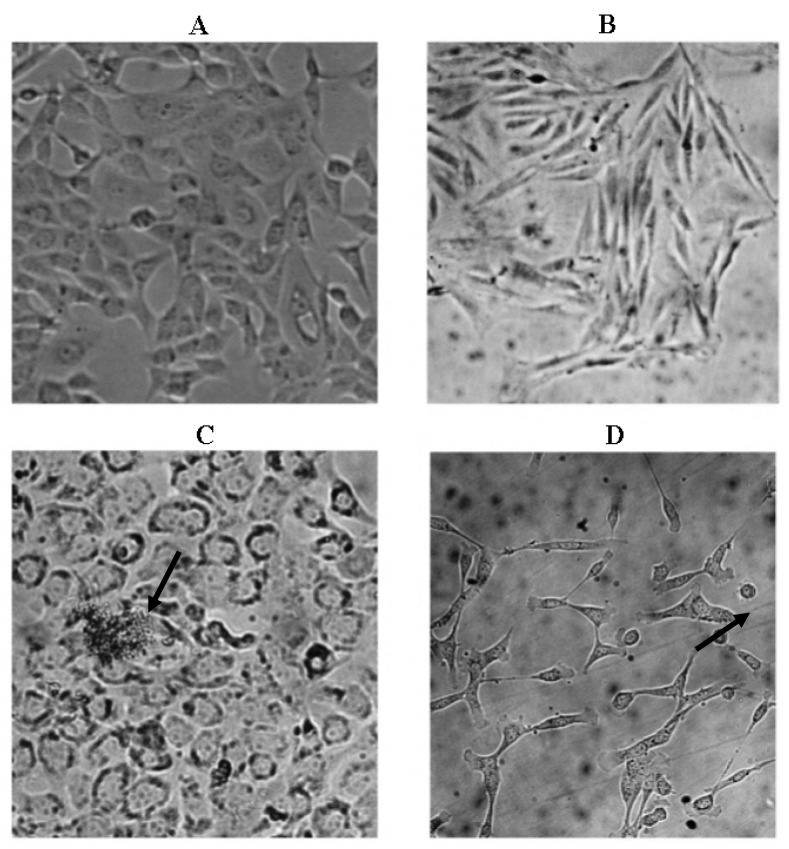
Morphological changes observed in Chang and Vero cell lines upon treatment with isolated PEs at CC_50_ concentration after 24 h incubation examined by light microscopy at 200× magnification. Chang (**A**: untreated; **C**: treated), Vero (**B**: untreated; **D**: treated).

The results of morphological changes visualized in both cell lines upon treatment with isolated PEs at CC_50_ concentration (C,D) after 24 h incubation are presented in Figure 4. Significant morphological changes, detachment and destruction of cells were observed upon treatment with PEs after 24 h incubation in both adherent cell lines.

According to these microscopic observations (Figure 4), cell damage resembled apoptosis as cell walls were not intact and apoptotic bodies were seen. The disruption in the integrity of the plasma membrane is one of the earliest features in apoptosis. Both cell lines displayed death upon treatment with the PEs at CC_50_ concentration at 24 h incubation. The cells subsequently detached completely over the following hours of incubation. The characteristics observed in this study were also reported by Bond *et al.*[15] who observed the detachment of human pancreatic adenocarcinomas cells upon 48 h exposure to PMA. Halaweish *et al*. [16] reported the changes in morphology, epidermal growth factor binding, arachidonic acid metabolite release and ornithine decarboxylase activity of the cells treated by plant ingenol and phorbol ester diterpenes.

### 2.4. Analysis of Apoptosis by Flow Cytometry

**Figure 5:**
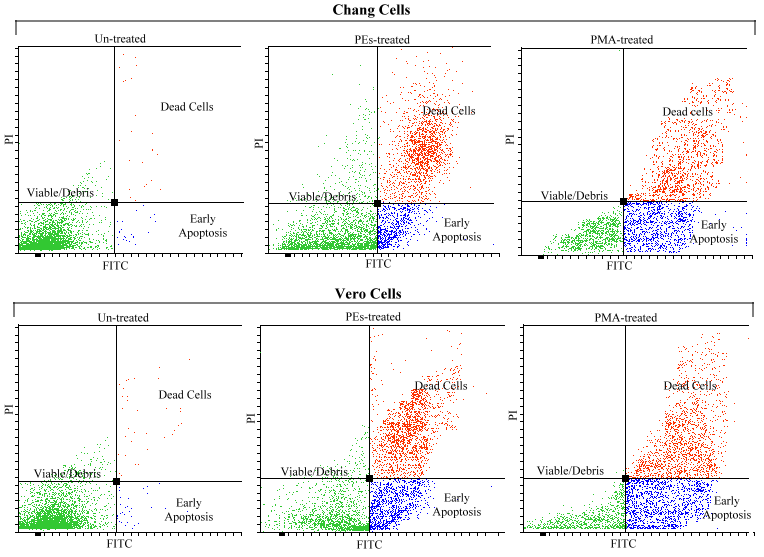
Flow cytometry analyses of Chang and Vero cells including untreated, PEs treated and PMA treated cells upon 24 h incubation.

In order to confirm the apoptosis cell death, the cells were double stained with FITC Annexin V and PI (propidium iodide) and subjected to flow cytometry. Figure 5 shows the flow cytometry analyses of both cell lines treated with PEs and PMA upon 24 h incubation. The flow cytometry results confirmed that PEs isolated from Jatropha meal and also PMA induced apoptosis cell death upon 24 h exposure.

As observed in Figure 5, the untreated cells showed the lowest intensity in the absorption of FITC Annexin V and PI dyes due to the viability of the cells and presence of intact cell membrane. The early apoptotic cells showed higher intensity in absorption of FITC Annexin V. The translocation of phosphatidylserine (PS) from the inner to the outer leaflet of the plasma membrane in the apoptotic cells, exposed the PS to the external cellular environment. The Annexin V labeled with a fluorescent FITC has a high affinity for PS. Thus, FITC Annexin V bound to the PS, indicating the cells undergoing apoptosis. The dead cells showed higher intensity in FITC Annexin V and PI as these dyes could easily pass through the disrupted cell walls and bound to the translocated PS and DNA respectively.

Table 2 shows the average of viable, apoptotic and dead cells percentage obtained from three independent experiments upon 24 h treatment analyzed by flow cytometry. These results showed that cells viability of Chang and Vero cell lines with values of 97.1% and 97.0% decreased significantly (*p* < 0.01) to 32.3% and 24.6% upon treatment with PEs and to 23.3 and 17.3 upon treatment with PMA respectively. The Chang and Vero cell lines showed 22.2% and 29.3% apoptotic cells upon treatment with PEs, while cells treated with PMA showed significantly (*p* < 0.01) higher values at 27.6% and 35.6%, respectively. These findings indicate that Chang cells were less prone to the effect of PEs and PMA as compared to the Vero cells and these results were consistent with the findings observed in the proliferation assay (Section 2.2). Although, the PEs appeared to be less active as compared to the PMA in induction of apoptosis, the percentage of dead cells indicated no significant difference between the cells treated with PEs and PMA. The difference in the potential of PMA and PEs in induction of apoptosis could probably due to the numbers or the position of functional groups present in PEs structures.

The role of PEs on induction of tumorgenesis, apoptosis, inflammation and survival of the cells depends on the type of activated PKC isozymes triggering different pathways. Indeed, emerging information suggests that PEs as PKC activators may induce apoptosis [6,7,17–19] or tumor formation [9,20,21]. The effects and mechanism of action of PEs depend on the types of phorbol ester, types of cell, time of exposure and the concentrations used. These factors may alter the balance between pro-apoptotic and anti-apoptotic pathways resulting in tumor or apoptosis of the cells.

### 2.5. DNA Fragmentation

**Figure 6:**
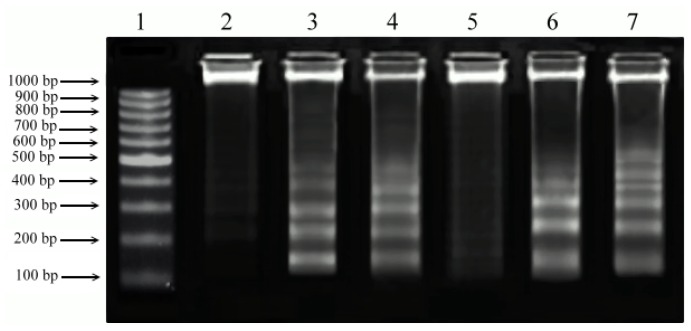
DNA fragmentation induced by isolated PEs and PMA in both cell lines at CC_50_ concentration. The extracted DNA was run on 2% agarose gel and the image was documented using Bio-Rad Gel documentation system. Lane 1: 1 kb DNA ladder; Lane 2: un-treated Chang; Lane 3: Chang + PEs; Lane 4: Chang + PMA; Lane 5: un-treated Vero; Lane 6: Vero + PEs; Lane 7: Vero + PMA.

The characteristics of apoptotic program involve certain morphologic features including loss of plasma membrane asymmetry and attachment, condensation of the cytoplasm and nucleus and internucleosomal cleavage of DNA. Isolated PEs and PMA induced nucleosome-size DNA fragmentation (Figure 6), a biochemical hallmark of apoptosis in the cells. The presence of DNA cleavage bands in cells treated with PEs indicated the similar cytotoxic effects of PEs to that of PMA. This result is in agreement with Day *et al*. [22] who observed changes in morphological features, apoptosis and endonuclease digestion of genomic DNA after 24 h incubation in LNCaP cells treated with PMA. Similarly, Rennecke *et al*. [23] have also found the DNA fragmentation in primary mouse thymocytes after prolonged treatment (>18 h) with PMA.

### 2.6. Western Blot Assay

**Figure 7:**
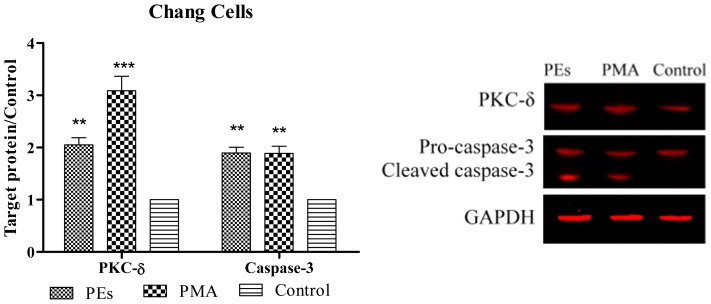
Expression of PKC-Δ and caspase-3 proteins in treated and untreated Chang cell line. Cells were treated with isolated PEs from *Jatropha* meal and PMA at the CC_50_ concentration incubated for 24 h. Equal amounts of total cellular protein of treated and control cells were subjected to Western blot analyses for PKC-Δ, caspase-3 and GAPDH protein expression. All values represent mean ± standard error from three independent experiments, ***p < 0.001 and **p < 0.01 indicate significant difference compared to the untreated control.

**Figure 8:**
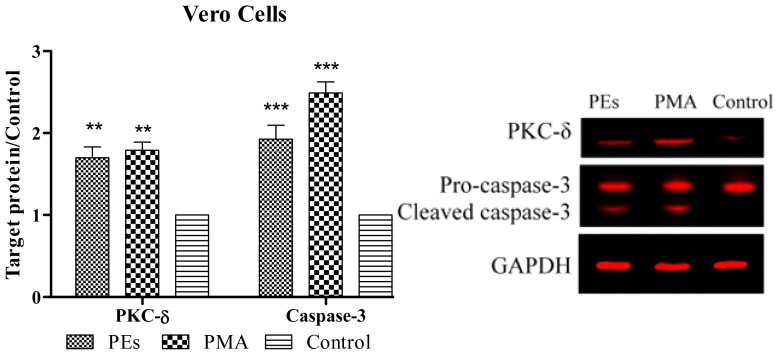
Expression of PKC-Δ and caspase-3 proteins in treated and untreated Vero cell line. Cells were treated with isolated PEs from Jatropha meal and PMA at the CC_50_ concentration incubated for 24 h. Equal amounts of total cellular protein of treated and control cells were subjected to Western blot analyses for PKC-Δ, caspase-3 and GAPDH protein expression. All values represent mean ± standard error from three independent experiments, ***p < 0.001 and **p < 0.01indicate significant difference compared to the untreated control.

As shown in Figures 7 and 8, PKC-Δ was significantly (*p* < 0.01) over-expressed in Chang and Vero cell lines upon 24 h treatment with isolated PEs and PMA. The results also showed the cleavage of caspase-3. The results were significantly (*p* < 0.01) higher than that of the control in both cell lines (Figures 7 and 8).

The individual roles of PKCs in the regulation of apoptosis have been reported in various cells. In most cells, PKC-α, ɛ and ι act as anti-apoptotic kinases, whereas PKC-β, μ and Δ act as pro-apoptotic kinases [24]. The over-expression of PKC-Δ observed in this study was in agreement with Fujii *et al*. [25] who observed the apoptosis in prostate cancer cells upon treatment with PMA which mediated through over-expression of PKC-Δ. Apart from that, Hatton *et al*. [8] also addressed the role of PKC-Δ in execution of caspase cascade proteins leading to apoptosis cell death.

In this study, activation of PKC-Δ probably led to the activation of caspase-3 protein. The observation of activated caspase-3 protein is considered as one of the hallmarks of apoptosis. The activation of caspase-3 protein in line with flow cytometry and DNA fragmentation results confirmed the apoptosis cells death when cells were treated with PEs and PMA. Similar observation was reported by Park *et al*. [26] who induced the apoptosis using PMA in gastric cancer cells through activation of PKC-Δ and finally execution of caspase-3 protein.

Phorbol esters exert a variety of effects in cellular systems that include proliferation, differentiation and cell death. The multiplicity effect of PEs on biological systems has been discussed by Rennecke *et al*. [23] and Harkin *et al*. [27]. Besides the type of PEs, another important factor is the time of exposure. They reported that, the anti-apoptotic effects of PEs in the cells are observed within the first 2–4 h upon treatment, while prolong exposure (>18 h) down-regulated the anti-apoptotic PKC species and up-regulated the pro-apoptotic PKC species.

The present study showed that isolated PEs from *Jatropha* meal initially disrupted the cell-substream adhesion without immediate loss of viability, subsequent cells detachment and finally death with apoptosis characteristics in Chang and Vero cell lines. This finding extended the information and revealed the cytotoxic effect of PEs as inhibitor of cell proliferation.

## 3. Experimental Section

### 3.1. Plant Materials

The *Jatropha curcas* L. plant was collected from the farm of Faculty of Agriculture, Universiti Putra Malaysia with the GPS location of 3°0′26.91″N latitude and 101°42′13.24″E longitude for identification by Mr. Shamsul Khamis. A voucher specimen (SK1764/2010) was deposited in the Phytomedicinal Herbarium, Institute of Bioscience, Universiti Putra Malaysia, Serdang, Selangor, Malaysia. Upon confirmation of the plant, the mature *J. curcas* seeds were collected from the farm, air dried and dehulled. The kernel were ground by using a mechanical grinder followed by oil extraction with Soxhlet apparatus, using petroleum ether (boiling point of 40–60 °C), for 16 h [28]. Defatted kernel (*Jatropha* meal) was air dried at room temperature and kept in screw cap bottle at −20 °C.

### 3.2. Phorbol Esters Isolation

Phorbol esters from *Jatropha* meal were isolated according to Li *et al*. [11]. Briefly, 4 g of *Jatropha* meal was extracted five times using methanol ground in pestle and mortar. The methanol was evaporated by rotary evaporator. The crude methanolic extract was dissolved in 5 mL of methanol and an aliquot was loaded on a high-performance liquid chromatography (HPLC) Waters Alliance 2695 Separations Module with a Waters 996 Photodiode Array Detector (Waters, Milford, MA, USA). It was equipped with the reverse-phase, C_18_ LiChrospher 100, 250 × 4 mm I.D and 5 μm pore size column (Agilent Technologies, Waldbronn, Germany). The separation was performed using a gradient elution with solvents comprising deionized water and acetonitrile [2,10]. The absorbance was read at 280 nm and phorbol esters were carefully collected using a fraction collector (Waters, Milford, MA, USA) at 24.4, 25.4, 26.4 and 26.9 min. The collected fractions were pooled and freeze dried. Isolated PEs were redissolved in dimethyl sulfoxide (DMSO) and analyzed by HPLC to determine the purity and concentration. The concentration of the isolated PEs used in this study was expressed as equivalent to a standard, phorbol-12-myristate 13-acetate (PMA).

### 3.3. Cell Lines and Cell Culture

The human hepatocytes (Chang ATCC: CCL-13) and African green monkey kidney cells (Vero ATCC: CRL-1586) were purchased from the American Type Culture Collection (ATCC). Cells were grown as monolayers in a T-75 cm^2^ culture flask. The Dulbecco’s Modified Eagle Medium (DMEM) was supplemented with 2.0 g/L sodium bicarbonate and 10% fetal bovine serum. The cell cultures were maintained in a humidified atmosphere of 5% CO_2_ at 37 °C and were harvested when they reached 80% confluency [29].

### 3.4. Proliferation Assay

Cell proliferation was determined using 3-(4,5-Dimethylthiazol-2-yl)-2,5-Diphenyltetrazolium Bromide (MTT) according to Sharif *et al*. [30]. Monolayers of the cells (5 × 10^3^/100 μL) were grown in 96-well microtitre plates and treated with the isolated PEs from 200 μg to 50 μg/mL by serial dilution. After 24 h incubation at 37 °C, cells proliferation assay was determined based on the reduction of MTT by the mitochondrial dehydrogenase of intact cells into an insoluble purple formazan product. Phorbol-12-myristate 13-acetate (PMA) (Sigma) was used as a positive control.

### 3.5. Microscopic Examination

Cells were cultured and treated at CC_50_ concentration with isolated PEs and positive control. Morphological apoptotic changes were examined after 24 h incubation and photographed using a phase-contrast microscope [31].

### 3.6. Analysis of Apoptosis by Flow-Cytometry

A fluorescent-activated cell sorting (FACS) analysis was performed to detect apoptosis. The Chang and Vero cells were seeded at the density of l × 10^6^ cells per 75 cm^2^ flask and cultured for 24 h in DMEM. Once confluent, the media were removed and fresh media containing PEs and PMA at the CC_50_ concentration were added. The treated cells were then incubated at 37 °C in 5% CO_2_ for 24 h. The FITC Annexin V Apoptosis Detection Kit I (BD Biosciences Pharmingen, San Diego, CA, USA) was used to stain the cell, following the manufacturer's instructions. The stained cells were monitored by flow cytometry (FACS-Canto II BD Biosciences) and the data were analyzed using Diva software (BD Biosciences). Cells that were considered viable would be FITC Annexin V and PI negative, while cells in early apoptosis would be FITC Annexin V positive and PI negative, and cells in late apoptosis or already dead would be both FITC Annexin V and PI positive.

### 3.7. DNA Fragmentation Assay

Different cell lines were treated at CC_50_ concentration of PEs and PMA and incubated for 24 h. Cultured cells were harvested and washed by PBS and pelleted by centrifugation at 300× *g* for 10 min. DNA was extracted from cells using extraction buffer containing Na_2_HPO_4_ and citric acid followed by addition of RNase and proteinase K according to Darzynkiewicz and Juan [32]. The extracted DNA was loaded on 2% agarose gel, electrophoresed at 50 V for 3 h and stained with ethidium bromide.

### 3.8. Western Blotting

Expression of protein kinase C-Δ (PKC-Δ) and caspase-3 proteins were assessed by Western blot analysis. Briefly, cells were trypsinized, harvested and washed three times with cold PBS. Then the cells were lysed in 100 μL of Lysis buffer (0.5% Triton X-100, 2 mM EDTA in 20 mM Tris-HCl pH 7.5) containing 10 μL/mL of Protease Inhibitor Cocktail (ProteoBlock Protease Inhibitor Cocktail, Fermentas, Glen Burnie, MD, USA) at 4 °C. Cells were then sonicated and incubated in ice for 20 min and supernatant collected after centrifuging at 14,000× *g* for 30 min. Protein concentration was determined in supernatant using the Protein Assay Kit (Bio-Rad Laboratories Inc., Hercules, CA, USA) and 20 μg of protein was denatured by incubation at 95 °C for 5 min and subjected to electrophoresis using Tris-glycine polyacrylamide gel. Proteins were transferred electrophoretically to a PVDF membrane using the Hoefer Semi-Dry Transfer Unit, (Hoefer Scientific Instruments, San Francisco, CA, USA). After electroblot transfer of the protein, membranes were washed twice and incubated with Odyssey Blocking Buffer (LI-COR, Lincoln, NE, USA) for 1 h at room temperature with rocking to block non-specific antibody binding. Then the membrane was incubated overnight with a 1:1000 dilution of PKC-Δ (PAB18258 Abnova), 1:1000 dilution of GAPDH (Thermo Scientific MA1-4711) and 1:500 dilution of Caspase-3 (Biorbyt orb10237) primary antibodies. Membrane was washed with 0.05% PBST (phosphate buffer saline and Tween 20) three times for 5 min. For detection with the Odyssey imaging system, a 1:10000 dilution of the IRDye 800 CW Goat Anti-Rabbit Secondary Antibody or-IRDye 680 Goat Anti-Mouse Secondary Antibody was used. Membrane was washed with 0.05% PBST three times for 5 min. The membrane was dried and visualized using the Odyssey Infrared Imaging System (LI-COR, Lincoln, NE, USA) and Odyssey software was used to determine the intensity of the proteins band.

### 3.9. Statistical Analysis

Statistical analysis was conducted using GLM procedure [33] using a complete randomized design following the model: *Yi* = *μ* + *Ti* + *ei*, where *μ* is the mean value, *Ti* is the treatment effect and *ei* is the experimental error, respectively. Differences in LSD were considered significant at *p* < 0.05. GraphPad Prism 5 software [27] was used for all the statistical analyses in Western blotting.

## 4. Conclusions

Phorbol esters isolated from Jatropha meal impaired the proliferation of human hepatocyte cells and kidney cells from African green monkey by induction of apoptosis. The PEs induced the over-expression of PKC-Δ and activation of caspase-3 proteins. The kidney cells appeared to be more susceptible to PEs as compared to the liver cells. These results were comparable to that of the PMA. Consequently, PEs present in Jatropha meal caused toxicity through apoptosis cell death and their complete removal is of prime importance when any dietary application of Jatropha meal is to be considered in the animal industry.

## Figures and Tables

**Table 1:** Cytotoxic concentration (CC_50_) of isolated PEs from *Jatropha* meal and PMA.

	CC_50_ μg/mL		
	
	Chang	Vero	S.E.M
**Isolated PEs**	125.9 ^a^	110.3 ^b^	2.79
**PMA**^1^	124.5 ^a^	106.3 ^b^	3.06

1 PMA: phorbol- 12-myristate 13-acetate; Analyses were done in triplicates; CC_50_: cytotoxic concentration that produced 50% cells death; Means within a row with different letter are significantly different (*p* < 0.01).

**Table 2:** Percentage of the viable, apoptotic and dead cells analyzed by flow cytometry.

	Chang Cells (%)			Vero Cells (%)			
			
	Un-treated	PEs-treated	PMA-treated	Un-treated	PEs-treated	PMA-treated	S.E.M
Viable	97.1 ^a^	32.3 ^b^	23.3 ^c^	97.0 ^a^	24.6 ^c^	17.3 ^d^	2.78
Apoptotic	1.6 ^d^	22.2 ^c^	27.6 ^b^	2.0 ^d^	29.3 ^b^	35.6 ^a^	2.28
Dead	2.0 ^b^	48.3 ^a^	51.6 ^a^	1.3 ^b^	50.6 ^a^	53.3 ^a^	3.47

Analyses were done in triplicates; At least 13,000 cells were analyzed by flow cytometry; Means with different superscripts within rows are significantly different (*p* < 0.01).
